# Inadequate Gonadal Replacement in Patients with Turner Syndrome may Result in Pituitary Volume Enlargement

**DOI:** 10.2174/1573405619666230223170130

**Published:** 2024-11-25

**Authors:** Gamze Akkus, İrem Kolsuz, Sinan Sözütok, Bilen Onan, Barış Karagun, Mehtap Evran, Murat Sert, Tamer Tetiker

**Affiliations:** 1 Faculty of Medicine, Division of Endocrinology, Cukurova University, Adana, Turkey; 2 Faculty of Medicine, Division of Internal Medicine, Cukurova University, Adana, Turkey; 3 Faculty of Medicine, Division of Radiology, Cukurova University, Adana, Turkey

**Keywords:** Turner syndrome, Hypogonadism, Hormone replacement therapy, Pituitary hyperplasia, Estrogen, MRI imaging

## Abstract

**Objectives:**

Patients with Turner syndrome need hormone replacement therapy for puberty induction. However, it is not known whether inadequate hormone replacement therapy affects the pituitary.

**Material and Methods:**

Patients with Turner syndrome (n=35) and healthy control (n=20) (age/gender matched) subjects were included. MRI imaging of the pituitary was used to calculate pituitary volumes. According to the estradiol regimen, patients were divided into two groups; (i) those treated with low-dose conjugated oestrogen (CE, 0.625 mg) and (ii) those treated with combination therapy (ethinyl estradiol+sipropterone acetate; 35 mcg/2 mg). Pituitary measurements were calculated according to pituitary borders and their distances to each other *via* pituitary MRI.

**Results:**

Pituitary hyperplasia (0.58±0.15 cm^3^ *vs*. 0.40±0.17 cm^3^) was determined in patients with low dose conjugated estrogen compared to the other patients or healthy control subjects (0.42±0.16 cm^3^) (p=0.005). Serum FSH levels of the patients treated with low dose CE were also higher compared to the patients who received combination therapy (p=0.001).

**Conclusion:**

Inadequate hormone replacement therapy can cause devastating effects on the bones and uterine health and disrupts the pituitary structure.

## 1 INTRODUCTION

Turner syndrome (TS) is a disorder of phenotypic females who have one X chromosome and complete or partial absence of the second X chromosome. It has been characterized according to physical features, such as neck webbing, short stature, and lymphedema. These patients also have ovarian insufficiency, congenital cardiovascular diseases, renal anomalies, and an increased risk of celiac diseases [1-3]. Turner syndrome is presented as hypergonadotropic hypogonadism due to gonadal dysgenesis and primary or secondary amenorrhea. A majority of prepubertal patients with ovarian failure and reduced feedback experience significantly elevated FSH and LH levels during early childhood (0-5 years) and adolescence (> 10 years). Since the specific karyotype of TS (X- monosomic forms) seems to be highly predictive of the remaining ovarian function, one-third of girls with mosaicism have spontaneous thelarche and rare menstrual cycles. Most patients with TS need convenient hormone replacement therapy and regular follow-up. The goal of optimal hormone is the induction of puberty and maintaining secondary sex characteristics or attaining peak bone mass [4-6]. It has been recommended that estrogen replacement should start between 11 and 12 years and increase until adolescence. Low-dose estradiol (0.25 mg /day-micronized oral E_2_ or 2 mcg/day –ethinyl estradiol) should be preferred in prepubertal ages [7]. In spite of all of these recommendations, the most suitable replacement therapy, timing of replacement, dosing, route of administration, and possible beneficial effects are still unclear.

To date, cardiovascular malformation or renal abnormalities in patients with TS have been researched, and many studies have been published. However, pituitary abnormalities are not common in these patients [8, 9]. In the literature, a few case reports of women with TS and pituitary micro or macroadenomas were reported [10-12]. In addition, pituitary hyperplasia is defined as a non-neoplastic increase in one or more functionally distinct types of pituitary cells [13]. Hormonal disturbances (severe hypothyroidism or adrenal failure) can cause pituitary hyperplasia. It is clearly documented by massive thyrotrope that hyperplasia can mimic tumors and compress adjacent tissues [14]. Additionally, the differential diagnosis of pituitary enlargement should be differentiated clearly from a pituitary adenoma. If a misdiagnosis is made, surgical removal in the case of pituitary hyperplasia will cause irreversible pituitary dysfunction and result in growth or neurocognitive dysfunction. Gonadotroph hyperplasia is a rare but well-recognized response to castration in animal models and humans with primary hypogonadism. Therefore, the occurrence of adenoma (micro or macro) or hyperplasia can cause confusion in clinical practice. Prolonged oestrogen deficiency in girls with TS throughout the critical phases of childhood may have detrimental effects on pituitary development. Apart from that, no studies elaborately investigate pituitary images in patients with TS. We aim to evaluate the pituitary volume of patients with Turner syndrome who received adequate hormone replacement therapy or did not receive this therapy. In addition, compartments of pituitary volumes in patients with TS and healthy control patients were reported in the current study.

## 2 MATERIALS AND METHODS

### 2.1 Subjects

Information on patients cytogenetically diagnosed with Turner syndrome was retrospectively collected from January 2010 to 2022 in our center. Turner syndrome is defined as a karyotype with a 45, X cell line (*i.e.,* a complete absence of X chromosome) or a structurally abnormal or absent short arm of the X chromosome. Karyotypes were generated from peripheral blood samples, and cytogenomic analyses were performed using G-banding. Abnormalities in the karyotype were presented based on the International System Cytogenomic Nomenclature (ISCN 2020) criteria [14].

Informed written consent was signed by the parents or legal guardians of the subjects or by the subjects. This study was approved by the Ethics Committee of the Faculty of Medicine, Cukurova University, Turkey (number 118).

### 2.2 Study Design

#### 2.2.1 Clinical Assessment

It was performed with special emphasis on the duration of hormone replacement therapy.

#### 2.2.2 Laboratory Assessment

1. Karyotype was obtained from patients’ records.
2. Serum levels of free thyroxine (T4), thyroid stimulating hormone (TSH), follicle-stimulating hormone (FSH), luteinizing hormone (LH), growth hormone (GH), estradiol (for women), cortisol, adrenocorticotropic hormone (ACTH), and prolactin (PRL) were assessed.

#### 2.2.3 Radiological Assessment

All patients with TS (n=35) were evaluated in terms of pituitary volumes on pituitary imaging of MRI. Pituitary imaging of (age and gender-matched) healthy controls (n=20) was selected randomly from our database system. The indications for pituitary imaging of healthy controls were headache (40%), neurological symptoms (30%), or other non-specific symptoms (30%).

#### 2.2.4 Exclusion Criteria

The exclusion criteria are as follows:

Patients with any pituitary disease, having a history of pituitary surgery, having a history of malignancy, and using drugs that affect the hypothalamic-pituitary axis.

#### 2.2.5 Selection of the Patients and Subgroups

From our hospital patient data system (ENLIL data system), we obtained information regarding the prescribed treatment for all patients. All patients were under hormone replacement therapy, but some of them failed to receive combination therapy (estrogen and progesterone),

Patients were divided into two groups as follows:

1. Patients were treated with only estradiol replacement therapy (conjugated estrogen 0.625 mg, n=20) and had plasma estradiol levels lower than 15 pg/mL.
2. Patients were treated with combination therapy (ethinyl estradiol+sipropterone acetate; 35 mcg/2 mg, n=15) and had plasma estradiol levels above 15 pg/mL.

All blood samples from patients were blindly analysed by the technician in the same laboratory for age, pubertal staging, and karyotype. Estradiol was measured by radioimmunoassay (Pantex, Santa Monica, CA).

### 2.3 MRI Techniques and Pituitary Volume Measurements

**Fig. (1):**
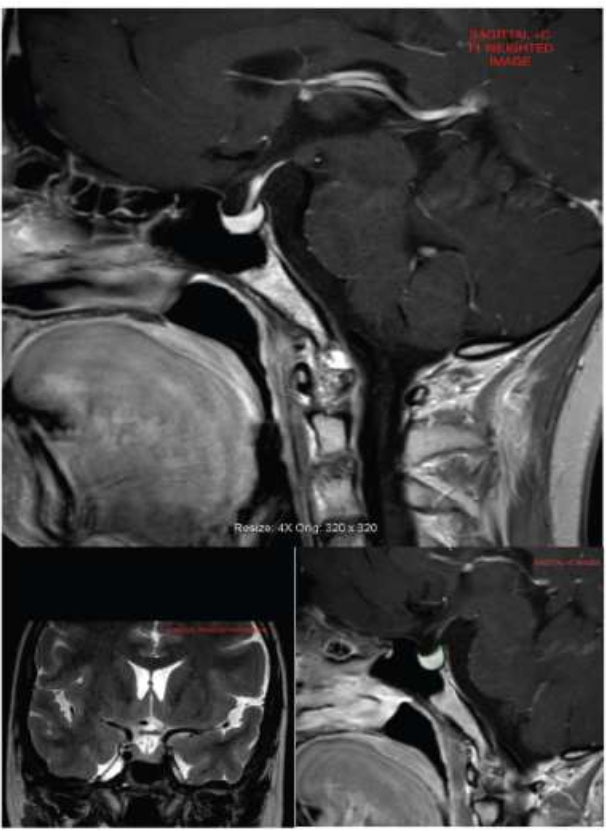
Coronal and Sagittal post-contrast image shows enlarged sella with marked of the pituitary gland of the stalk.

Diameters of the pituitary gland were measured by using pituitary MRI according to the pituitary borders and their distances. The MRI images were performed by two expert neuroradiologists with >5 years of experience in neurological radiology. A 3.0 Tesla scanner (Philips Achieva, Philips Medical Systems, Best, The Netherlands was used for patients for pituitary measurements. T1 sagittal contrast-enhanced images were used for volumetric evaluation in Osirix Dicom Viewer (Pixmeo SARL, Geneve, Swiss) (Fig. **1**).

### 2.4 Statistical Analysis

While categorical variables were demonstrated as numbers and percentages, continuous variables were clarified as mean and standard deviation and median and minimum-maximum. A chi-square test was performed to compare categorical variables between the groups. The normality of distinction for continuous variables was evaluated by the Shapiro-Wilk test. The Student's t-test or Mann-Whitney U test was used for determining continuous variables. Pearson Correlation Coefficient or Spearman Rank Correlation Coefficient was used for evaluating the correlations between the measurements. All analyses were performed by using IBM SPSS Statistics Version 20.0 statistical software package. The statistical level of significance for all tests was considered to be 0.05.

(SPSS reference: IBM Corp. Released 2011. IBM SPSS Statistics for Windows, Version 20.0. Armonk, NY: IBM Corp.)

## 3 RESULTS

The mean age of the patients with TS and healthy controls was 25.9 ± 9.6 *vs*. 30.6 ± 10.2, respectively (p=0.102). The karyotype distribution of the patients with TS is shown in Table **1**.

Mean FSH, LH, and estradiol (E2) levels of the patients with TS were 63.2 ± 51.8 (range 0.1-191 mIU/mL), 20.2 ± 18.8 (range 0.02-69.8 mIU/mL), and 51.7 ± 125.4 pg/mL, respectively.

Regarding hormone replacement therapy, 20 of the patients were treated with low-dose conjugated oestrogen (CE, 0.625 mg), and the others (n=15) were treated with combination therapy (ethinyl estradiol+sipropterone acetate; 35 mcg/2 mg). Estradiol levels of patients with low dose CE were lower (<15 pg/mL) than the patients with combination therapy (>15 pg/mL). Serum FSH levels of the patients treated with low-dose CE were higher than the patients treated with combination therapy (p=0.001). Bone mineral density measurements of the patients with low dose CE were also lower than the other group (p=0.027) (Table **2**).

When we compared the pituitary volumes of all patients and healthy control subjects, volumes in patients treated with low dose CE (0.58 ± 0.15 cm^3^) were higher than in patients treated with combination therapy (0.40 ± 0.17 cm^3^) or healthy controls (0.42 ± 0.16 cm^3^) (p=0.005).

Osteoporosis was also more common in patients treated with low dose CE (estradiol <15 pg/mL) than the patients treated with combination therapy (estradiol>15 pg/mL, p=0.027).

## 4 DISCUSSION

The study results showed that pituitary hyperplasia correlates with inadequate hormone replacement therapy in patients with TS. Hormone replacement therapy is generally aimed at sustaining feminization but also at preventing systemic alterations in estrogenic deficiency.

Most patients with TS need hormonal therapy to induce puberty and maintain female secondary sex characteristics. The clinical spectrum of hormone replacement therapy should be started between 11 and 12 years of age and increased as an adult dosage over a period of 2-3 years. This theoretically translates that an optimum dose (10-20 mc/day ethinyl estradiol or 1-4 mg/day conjugated estrogen) of estrogen is recommended for adult patients, and this treatment should be continued until the age of menopause [7].

In addition to maintaining secondary sex characteristics with an optimum dose of hormonal therapy, inadequate replacement therapy has been related to metabolic alterations (increased fasting plasma glucose and low-density lipoprotein levels), increased risk of osteoporosis or negative effects on the quality of life (QoL) [15-18].

Many previous studies have shown that delaying treatment and insufficient doses of estrogen regimens could be deleterious to bones and uterine health and create hypogonadism effects. However, no studies investigated inadequate gonadal replacement treatment on the pituitary volume of patients with TS. Moreover, the general opinion is that pituitary abnormalities are not common in these patients. Until the present time, only case reports of patients with TS and pituitary adenomas have been presented in the literature. In our study, pituitary volumes in patients treated with low-dose conjugated estrogen (0.625 mg) treatment were higher than the patients treated with combination therapy (ethinyl estradiol+sipropterone acetate; 35 mcg/2 mg) (p=0.005). Since age is a confounding factor for pituitary volume, both groups were matched in terms of age. Although Scheithauer *et al*. [19] reported that gonadotroph hyperplasia was not seen in their postmortem case series, long-term hypogonadism due to inadequate hormone therapy explains the rebound gonadotroph cells hyperplasia.

Routine monitoring of LH and FSH is recommended during the optimum dosage of estrogen for the follow-up of patients with TS [20]. Previous studies [21, 22] have reported that serum gonadotropin levels were correlated with serum estradiol levels (with or without replacement therapy), specific karyotype, and remaining ovarian function. In our study, a majority of the patients had 45,X (n=28, 80%) and poor ovarian reserve. Since these patients treated with low-dose CE did not receive sufficient hormonal therapy, serum FSH levels (77.3 ± 49.9 *vs*. 15.7 ± 21.3, p=0.00 were much higher than those treated with combination therapy. This result also suggests that inadequate hormone therapy in patients with TS can trigger the pituitary gland, especially hyperplasia of gonadotroph cells.

Based on previous studies, the question of whether the simultaneous occurrence of Turner syndrome and pituitary hyperplasia is causal or incidental cannot be answered. Untreated hypothyroidism or Addison disease related to diffuse and nodular TSH or ACTH hyperplasia is demonstrated in published immunological and histological studies [6, 23]. It is also possible that gonadotroph cell proliferation is triggered under the continuous stimulus of the gonadotroph receptors due to inadequate therapy. In this study, patients with an optimum dose of estrogen replacement therapy had similar pituitary volumes to the healthy control group.

## CONCLUSION

In conclusion, the timing, route, and dose of hormone replacement therapy in patients with TS are controversial issues. Although a low dose of hormone therapy is recommended as an initial therapy, higher doses of estrogen therapy are required for adult patients with TS. It is important to keep in mind that the recommendation for estrogen replacement is for a deficient state and not to supplement endogenous hormones. Inadequate hormone replacement therapy can cause devastating effects on the bones and uterine health and also disrupts the pituitary structure.

## Figures and Tables

**Table 1:** Karyotype distribution among TS patients.

**-**	**N**	**%**
45,X	28	80
45,X(%33)/47,XXX(%66)	1	2.85
45,X(%80) / 46,X,+Xp(%20)	2	5.71
45,X/46,X,del(X)(q24)	2	5.71
45,X0 (85)/46,XY(15)	1	2.85
46,X delXq27.1	1	2.85

**Table 2:** Comparement data of the patients with estradiol levels below or above 15 turner syndrome.

**-**	**Estradiol>15** pg/mL* **(n=15)**	**Estradiol<15** pg/mL* **(n=20)**	**p**
**Age (year)**	22.7±4.3	26.8±10	0.2
**FSH (mIU/mL)** **(1.27-19.26)**	15.7±21.3	77.3±49.9	0.001
**LH (mIU/mL)** **(1.24-8.62)**	22.6±21.3	19.5±12.4 19.5 19.5	0.86
**Prolactine (ng/mL)** **(2.64-13.13)**	16.1±9.3	13.9±9.3	0.45
**TSH (mIU/L)** **(0.38-5.33)**	2.2±1.2	2.7±1.6	0.34
**GH (ng/mL)** **(0-1)**	0.5±0.7	0.3±0.6	0.72
**ACTH (pg/mL)** **(10-60)**	12.2±0.7	14.4±6.6	0.69
**Cortisol (mcg/dL)** **(9-22)** **(6.7-22.6) (10-50)**	18.8±3.9	13.1±7.7	0.485
**Dexa (Z score)**	-2.5±0.9	-3.0±2.0	0.027
**Pituitary volume (cm^3^)**	0.40±0.17	0.58±0.15	0.005
**Hormone replacement therapy**	Ethinyl estradiol+ Cypropterone asetat (35 mcg/2 mg)	Conjugated estrogen (0.625 mg)	-

**Note:** *: referance range. Postmenauposal period: 15-40 pg/mL. Follicular phase: 27-122 pg/mL. Midcycle period: 95-423 pg/mL. Luteal phase: 49-291 pg/mL. FSH: Follicle Stimulating Hormone, LH: Luteinizing Hormone, TSH: Thyroid Stimulating Hormone, ACTH: Adrenocorticotroph hormone, GH: Growth Hormone.

## Data Availability

All the data and supportive information are provided within the article.

## LIST OF ABBREVIATIONS

ACTH: Adrenocorticotropic hormone
CE: Conjugated oestrogen
FSH: Follicle-stimulating hormone
GH: Growth hormone
LH: Luteinizing hormone
PRL: Prolactin
QoL: Quality of life
T4: Serum levels of free thyroxine
TSH: Thyroid-stimulating hormone

## STANDARDS OF REPORTING

STROBE guidelines were followed.
